# Educating sports managers for the sport industry 5.0 era: synergy of digital competencies and human-centric approach in the labor market

**DOI:** 10.3389/fspor.2026.1930990

**Published:** 2026-08-21

**Authors:** Daniel Opelík, Josef Voráček, Michaela Kaprálková

**Affiliations:** Department of Sport Management, Faculty of Physical Education and Sport, Prague, Czechia

**Keywords:** digital transformation in sport, employability, higher education, sport management, sport management education

## Abstract

**Objective:**

This study aims to analyze emerging labor market demands for sports managers in the context of Industry 5.0 and to evaluate the current level of graduates' professional preparedness, with a specific focus on the integration of digital competencies and a human-centric approach.

**Methods:**

A qualitative research design was employed using semi-structured interviews with two stakeholder groups: sport organization representatives (*n* = 17) and sport management graduates (*n* = 11). The study followed a hybrid deductive–inductive analytical approach supported by MAXQDA software. Thematic development was based on open, axial, and selective coding, while credibility and dependability were strengthened through triangulation, memoing, and an audit trail.

**Results:**

The findings reveal a fundamental shift in competency requirements within the Industry 5.0 environment. While graduates demonstrate adequate theoretical knowledge, they lack the ability to strategically integrate digital tools into managerial practice. Significant deficiencies are identified in soft skills, particularly in accountability, resilience, and communication. The study also highlights a persistent theory–practice gap and emphasizes the growing importance of experiential learning, international exposure, and continuous professional development.

**Conclusion:**

The study concludes that future sports managers must develop a synergistic combination of digital literacy and human-centric competencies. Bridging the gap between academic education and labor market demands requires a transformation of educational models toward experiential learning and the systematic integration of digital tools into curricula. These changes are essential to ensure long-term adaptability and employability in the evolving sports industry.

## Introduction

In contemporary society, sport represents not only a significant cultural and social phenomenon but has also become an integral component of the global economy. This modern sporting environment has long been characterized by ever-increasing complexity, professionalization, and globalization (1, 2). Consequently, there are rising demands for the effective management and organization of sports clubs, federations, and both local and global events, thereby continuously elevating the importance of sports managers as central figures within this ecosystem. Recent research further highlights that sport organizations operate in increasingly complex environments shaped by digital communication, stakeholder expectations, sustainability imperatives, and societal change (3).

The role of the sports manager is expanding dynamically, currently encompassing a wide spectrum of activities ranging from strategic planning and team leadership to sports event management, marketing, public relations, and the implementation of advanced technologies. A successful sports manager must therefore be capable of integrating insights from a multitude of disciplines, including management, economics, law, psychology, and information technology, while fully respecting the unique nuances of the sports environment. This requirement is consistent with person-environment perspectives, which emphasize the alignment between individual competencies and the evolving demands of the organizational and technological environment (4). It is no longer merely a matter of overseeing day-to-day operations; rather, it demands an organization's capacity to respond flexibly to dynamic shifts in the external environment, which is currently defined by rapid digitalization and technological advancement.

A pivotal turning point in this evolution is the advent of the Industry 5.0 era, which introduces the imperative to align technological innovation with human-centricity, sustainable development, and social responsibility within the sector. Alongside the ongoing digitalization and automation of sports services (5), managers are transforming from mere hierarchical supervisors into primary change agents, drivers of organizational adaptation (6–8), and key actors in the implementation of innovative strategic decisions.

The educational framework represents the critical factor determining whether these professionals will be equipped to face new sectoral and technological challenges. Academia confronts the formidable task of reflecting the turbulent development of technology alongside societal trends (such as inclusion or sustainability), adapting higher education programs to ensure that graduates possess not only theoretical knowledge but also the necessary practical agility. Only an educational system that remains profoundly open to innovation can secure the long-term competitiveness of the sports industry.

Although sports management degree programs are primarily designed to prepare graduates for the actual labor market, a noticeable discrepancy between academic education and the real-world demands of employers has been evident (9–11). To achieve successful employability in today's labor market, graduates are required to exhibit a significantly higher degree of adaptability and a commitment to continuous engagement in lifelong learning (12–15). Currently, this mismatch manifests most prominently in the absence of core competencies that bridge technological and human factors. The labor market now considers it imperative for sports managers to possess a high level of digital competencies (16–18), navigating areas such as social media, e-commerce, and project management, while effectively utilizing artificial intelligence tools (19–21). Concurrently with this emphasis on technology and digitalization, an expert understanding of human dynamics, soft skills, and communication proficiency is also demanded, as the full potential of technology cannot be effectively integrated into modern human teams without these capabilities.

Despite the growing scholarly attention devoted to digital transformation, employability, and competency development in sport management education, the existing literature still tends to examine these dimensions separately. Previous studies have addressed graduate preparedness, sport management curricula, digital literacy, internships, and labor market expectations, yet less attention has been paid to how technological competencies and human-centric managerial capabilities interact as an integrated readiness condition in the context of Industry 5.0. In particular, there remains limited empirical evidence combining the perspectives of sport industry employers and recent graduates to explain how digital tools, artificial intelligence, interpersonal competencies, accountability, and experiential learning jointly shape employability in contemporary sport organizations. This gap is important because Industry 5.0 does not merely require additional digital skills, but a socio-technical form of readiness in which technology is meaningfully embedded in human judgment, communication, ethical responsibility, and organizational practice.

The objective of this article is to comprehensively analyze these emerging labor market demands imposed on sports managers and to uncover the current state of their professional preparedness, focusing specifically on the intersection of digital innovation and the human-centric approach. By evaluating the strengths and weaknesses of graduates at the crossroads of technological readiness and soft skills, this study contributes to the ongoing discourse on shaping the future of sport. Rather than treating digital literacy and soft skills as separate competency domains, the study conceptualizes graduate readiness as a socio-technical capability: the ability to use AI-enabled and digital tools while preserving accountability, critical judgment, communication, resilience and ethical sensitivity. Based on these findings, concrete recommendations for educational curriculum innovation are presented in the conclusion, thereby helping to align the preparation of managers with the core values and imperatives of Industry 5.0.

## Literature review

Sports management as an academic discipline and professional practice has evolved significantly over recent decades, driven by the intense commercialization and professionalization of the broader sports industry (2, 20, 22). Managing sports organizations in the 21st century fully demands the application of sophisticated techniques and strategic frameworks commonly utilized within modern commercial enterprises (23, 24). Within the context of Industry 5.0, however, it is essential to intertwine these managerial techniques with human dynamics and technological innovations. The theoretical framework of this article rests upon four foundational pillars: the human-centric approach, the transformation of managerial roles, contemporary competency models, and the unique specificities of the sports labor market.

### The shift toward a “human-centric” approach in sports management

While earlier conceptualizations of management were frequently grounded in a purely rational-economic paradigm, contemporary perspectives prioritize social aspects and the human factor (25–27). This shift is particularly pronounced within the sports sector, given that sport cannot be understood merely as an industrial branch or a purely commercial activity; rather, it constitutes a phenomenon centered primarily around athletes and active communities (1, 28, 29). Value in sport is not mechanically generated by an organization alone; instead, it is co-created by a diverse network of actors (30, 31), including fans, volunteers, and non-profit entities.

For this reason, so-called human and interpersonal skills are gaining increased prominence. These skills, rooted in psychology, sociology, and ethics, are indispensable for sports managers across all levels of governance (16, 32). On a daily basis, sports managers must interact with a wide array of individuals across diverse socio-economic statuses, spanning from unpaid volunteers and interns to professional athletes and executives (33, 34). The capacity to effectively navigate this diversity, motivate colleagues through periods of both success and adversity (35), and cultivate a sustainable organizational culture (36, 37) has emerged as a core managerial capability.

### The transformation of managerial roles and functions driven by technology

The advent of advanced technologies and artificial intelligence (AI) is dynamically altering traditional managerial functions, namely planning, organizing, staffing, leading, and controlling (38, 39). Today, technology penetrates these core functions at every organizational tier. At the executive management level, the role is shifting away from mere operational oversight toward steering the future direction and long-term development of the organization (40, 41). This shift is deeply connected to strategic choices regarding digitalization and capital investments in the technological infrastructure of sports organizations. Amidst the proliferation of innovations and AI systems, the responsibility of executive leadership is to define a sustainable path for the organization, ensure transparent change communication, and provide a secure environment for experimenting with novel tools at lower levels of the hierarchy (42–44).

According to classical frameworks (e.g., Mintzberg), managers fulfill interpersonal, informational, and decisional roles (45–47). Within the digital era, the informational role (data monitoring and dissemination) and the decisional role (resource allocation for technological advancement) gain particular importance. Middle management increasingly acts as an information synthesizer, frequently processing insights generated by data and AI models, and emerges as a critical actor in executing strategic changes (48, 49) and nurturing innovations that sprout from the daily operations managed by frontline oversight.

### The synergy of digital and soft skills

The traditional approach to teaching and learning, which relied on a fixed set of knowledge and theories, is no longer sufficient (50, 51) for the modern labor market. Current competency models for sports managers are divided into two main streams that must operate in synergy (52–55):

- Generic Competencies: These encompass personal and social competencies, including effective personal time management, adaptability, the capacity to assume responsibility, teamwork and leadership skills, conflict resolution, and argumentation. Recently, these have been augmented by methodological competencies, such as analytical skills, information management, and the ability to navigate unexpected crisis situations (52).
- Sports-Specific Competencies: In addition to knowledge of sports policy, economics, and law, an unprecedented emphasis is now placed on digital literacy (17). Consequently, specific sports competencies are being enriched by the digital sphere. Sports organizations must require skills associated with operating within modern software platforms (17, 19, 56, 57), familiarity with e-commerce, expert social media management, and the ability to leverage artificial intelligence tools.

However, mere technological proficiency is insufficient on its own. Technology must be managed and implemented by sports managers who possess high levels of critical thinking, creativity, and the ability to communicate innovations across teams.

### Specifics of the sports labor market and the widening theory-practice gap

The application of the theoretical frameworks encounters the unique specificities of the sports labor market. This market is characterized by a high degree of volunteerism (58), immense dynamics, and globalized labor mobility (59, 60). Although educational institutions strive to produce career-ready graduates, scholarly literature has long identified a noticeable discrepancy (10, 11) between academic education and the actual demands of employers.

Employers report inconsistencies between the theoretical skills offered by graduates and the practical, agile skills realistically required by the modern sports market (21). Demand is growing rapidly precisely at the intersection of digitalization and a human-centric approach, namely in project management, online community engagement, and the utilization of AI tools, while simultaneously maintaining an understanding of sports culture and human motivation (1, 61, 62). Bridging this gap by integrating hard technological skills with soft human competencies thus becomes a central theoretical imperative for innovating the education of sports managers.

Taken together, the reviewed literature demonstrates that sport management education is increasingly expected to respond simultaneously to digital transformation, employability pressures, and the human-specific character of sport organizations. These research streams remain only partially integrated. Studies on digital literacy and artificial intelligence (17, 19, 63) tend to emphasize technological preparedness, while research on employability and sport management competencies (16, 52, 64) often highlights internships, communication, leadership, and practical experience. What remains insufficiently developed is a more integrative explanation of how these domains operate together in the everyday work of sport managers. This study therefore does not merely add another list of desirable competencies; rather, it examines how digital acceleration, human-centric competencies, experiential learning, and career sustainability form an interdependent readiness structure for sport managers in the Industry 5.0 era.

## Methods

The present study employs a qualitative research design to facilitate a profound understanding of the investigated phenomenon, uncover underlying relationships, and provide a detailed analysis of soft data associated with the professional preparedness of managers. Qualitative inquiry was deemed appropriate given the exploratory nature of the research problem and its focus on meanings, experiences, and contextual factors rather than quantifiable relationships.

The study follows hybrid deductive-inductive logic. An initial conceptual framework informed by the literature guided the development of the interview protocol and structured the inquiry into four thematic domains: education, internships and practical experience, career readiness, and skills. At the same time, openness to emergent themes was maintained throughout data collection and analysis, allowing for inductive refinement of the framework.

To enhance the rigor and trustworthiness of the study, multiple strategies were employed. Investigator triangulation was ensured by continuously consulting the research design, interview protocol, and analytical procedures with two independent experts in sport management. Theoretical triangulation was applied by interpreting findings through multiple conceptual perspectives related to employability, human capital, and labor market dynamics. Additionally, the research design incorporated a dual-perspective approach, examining the phenomenon from both the demand side (employers) and the supply side (graduates), thereby strengthening the explanatory depth and analytical robustness of the findings.

### Research questions

To guide the empirical inquiry, the study addresses the following research questions:

**RQ1:** What competency demands do sports management labor market stakeholders identify in graduates within the context of advancing technological integration?

**RQ2:** What educational model innovations are required to bridge the gap between academic preparation and professional practice, enabling graduates to effectively integrate emerging technologies and artificial intelligence tools into the management of sports organizations?

### Research sample

Semi-structured interviews served as the primary data collection method. The research sample was constructed using purposive sampling, complemented by intensity sampling to select information-rich cases relevant to the research objectives. A sequential recruitment strategy was employed, with participants added iteratively until theoretical saturation was reached, defined as the point at which no substantively new themes emerged from the data.

Theoretical saturation was assessed iteratively during data collection and preliminary analysis. After each set of interviews, newly emerging codes and categories were compared with the existing coding structure. Recruitment was concluded when additional interviews no longer produced substantively new categories but primarily confirmed or refined already identified patterns related to digital competencies, human-centric skills, practical preparedness, and career sustainability.

The final sample (*n* = 28) comprised two stakeholder groups. The demand side included sport organization representatives (*n* = 17; R1–R17), specifically managers and executives across different segments of the sport industry (e.g., professional clubs, federations). Participants were identified through expert consultation and professional networks to ensure relevance and diversity of perspectives. The supply side consisted of sport management graduates (*n* = 11; A1–A11) who completed their studies between 2016 and 2023 and were selected to reflect variation in early career trajectories.

The inclusion criteria for sport organization representatives were direct involvement in managerial, executive, coordination, or decision-making activities within sport organizations and sufficient professional experience to evaluate the preparedness of sport management graduates entering the labor market. The graduate group included individuals who had completed a sport management-related degree and had already gained initial professional experience allowing them to reflect retrospectively on the relevance of their academic preparation. This sampling strategy was selected to capture both employer-side expectations and graduate-side transition experiences, rather than to achieve statistical representativeness. Participants were therefore treated as information-rich cases capable of providing insight into the alignment between higher education and contemporary sport labor market requirements.

### Qualitative data collection

Data were collected through individual semi-structured interviews, with the data collection process completed in early 2025. The interview guide was developed based on the conceptual framework and pilot-tested with two respondents to ensure clarity and relevance. The semi-structured format enabled consistency across interviews while allowing flexibility for probing and the exploration of emergent themes.

Interviews were conducted either face-to-face at participants' workplaces or online via Microsoft Teams, depending on availability and preference. All interviews were audio-recorded with prior informed consent and subsequently transcribed verbatim. Interview duration ranged from 32 to 69 min for industry representatives and from 27 to 62 min for sport management graduates. The researchers remained attentive to reflexivity throughout the data collection process, acknowledging their professional background in sport management and its potential influence on data interpretation. Field notes were taken after each interview to capture contextual observations and preliminary analytical reflections.

### Data analysis

All audio recordings were transcribed verbatim and lightly edited to remove filler words without altering semantic meaning. The transcripts were imported into MAXQDA (version 24) software for qualitative data analysis. First, transcripts were read repeatedly to ensure familiarization with the data. The analytical process followed a hybrid approach, utilizing the constant comparative method across three sequential phases:

- Open Coding: The text was fragmented into discrete meaning units (words or sentences) and assigned descriptive codes, including *in vivo* codes derived directly from the participants' terminology. Analytical and methodological memos were generated throughout to document emerging insights and support transparency.
- Axial Coding and Categorization: The initial codes were compared, refined, and grouped into higher-order categories based on conceptual similarity and relationships between phenomena. To preserve sensitivity to stakeholder-specific perspectives, coding was initially conducted separately for each group (graduates vs. employers) before being integrated.
- Selective Coding and Framework Development: In the final phase, the refined categories were integrated into a core narrative skeleton. This step allowed for the conceptualization of causal and logical linkages between categories, translating the structured data into a coherent explanatory framework for scientific interpretation.

The four final themes were derived through an iterative process of comparing lower-level codes across both stakeholder groups and identifying recurring conceptual linkages. Initial codes related to artificial intelligence, project management platforms, social media, and digital tools were gradually integrated into the broader theme of technological integration and digital acceleration. Codes referring to accountability, feedback, communication, resilience, teamwork, and leadership were grouped under human dynamics and the soft skills deficit. Statements concerning insufficient practical experience, internships, and the transition from university to employment formed the theme of the theory–practice gap and experiential learning imperative. Codes related to international experience, intercultural competence, and lifelong learning were consolidated into the theme of career sustainability. The final thematic structure therefore emerged from the repeated comparison of participant accounts, rather than from a purely pre-defined coding scheme.

To enhance the transparency and dependability of the analytical process, selected stages of coding and category development were subject to internal discussion within the research team. In particular, the emerging coding framework and interpretation of key themes were reviewed collaboratively, allowing for the clarification of ambiguous coding decisions and the refinement of category definitions. This iterative process contributed to greater analytical consistency while acknowledging the interpretative nature of qualitative research. An audit trail documenting coding schemes, memos, and analytical decisions was maintained throughout the research process.

Several additional procedures were used to strengthen the trustworthiness of the analysis. Credibility was supported through the comparison of two stakeholder perspectives and by grounding the interpretation in representative quotations from both employers and graduates. Dependability was enhanced through the systematic documentation of category revisions and analytical memos in MAXQDA. Confirmability was addressed by discussing emerging interpretations within the research team to reduce the risk of individual interpretative bias. Although qualitative analysis inevitably involves researcher interpretation, these procedures were intended to make the analytical process transparent, traceable, and analytically coherent.

### Ethical considerations

This study was conducted in full compliance with the ethical standards required for academic research involving human participants and was formally approved by the relevant institutional Ethics Committee. Prior to data collection, all participants were provided with comprehensive information regarding the study's objectives, data handling procedures, and their right to withdraw from the research at any stage without penalty. Written informed consent was obtained from all respondents. To guarantee confidentiality and privacy protection in compliance with GDPR standards, all datasets were rigorously anonymized. Any potentially identifying institutional or personal details were removed. Data were securely stored and accessed only by the research team.

## Results

The qualitative data analysis, derived from semi-structured interviews with industry employers and sports management graduates, reveals a prominent shift in professional competence requirements within the era of Industry 5.0. Through the coding process and thematic analysis, four primary cross-cutting themes emerged as critical determinants of sports managers' readiness for the contemporary labor market: (1) technological integration and digital acceleration, (2) human dynamics and the soft skills deficit, (3) the misalignment between academia and market reality, and (4) Career sustainability. To enhance the transparency of the qualitative findings, Table 1 summarizes the four final themes, their main subthemes, and representative quotations illustrating the empirical basis of the thematic structure.

**Table 1:** Final themes, subthemes, representative quotations, and stakeholder sources.

**Theme**	**Main subthemes**	**Representative quotation**	**Stakeholder source**
Technological integration and digital acceleration	AI tools; project management platforms; social media; digital use	“Whoever learns to work with this highly proficiently will benefit from it and accelerate their efficiency.”	Employer R14
Human dynamics and the soft skills deficit	Accountability; resilience; communication; leadership	“Finding an individual who will actually accept that responsibility and is willing to bear it is, in my opinion, becoming harder and harder.”	Employer R8
Theory–practice gap	Practical readiness; internships; transition to work	“I think that is an irreplaceable experience. It gives a person more than certain courses do.”	Graduate A2
Career sustainability	International experience; intercultural competence; lifelong learning	“As for networking—well, I consider that to be the alpha and omega of career success.”	Graduate A8

Respondent labels identify stakeholder group and interview number.

These findings suggest that although theoretical preparedness is well-established, graduates face significant barriers when applying this knowledge within the digitized, highly dynamic environment of modern sports organizations.

### Technological integration and digital acceleration

The first core thematic area pertains to technological proficiency, which, within the context of Industry 5.0, is no longer perceived as a competitive advantage but rather as a necessity. Data analysis revealed that although graduates possess basic user literacy, they demonstrate a so-called “limited digital competence” upon entering the labor market. The underlying issue does not stem from an inability to operate technology, but rather from a deficiency in strategically integrating these tools into managerial processes.

Artificial Intelligence (AI) has emerged as a prominent phenomenon altering the nature of a sports manager's work. Industry representatives view AI tools as indispensable productivity accelerators. Proficiency in working with these systems (e.g., ChatGPT, Copilot) drastically streamlines both routine and creative processes. Respondent R14 explicitly stated: “*Whoever learns to work with this highly proficiently will benefit from it and accelerate their efficiency.”* This reality is also reflected by the graduates themselves, who have already implemented AI into their professional practice. Graduate A4 described the benefits of AI in daily operations as the ability to “*save time regarding creative tasks, or precisely writing emails, general communication, brainstorming copywriting, and similar things.”* These accounts indicate that AI is increasingly perceived not only as a technological innovation, but as a practical instrument for accelerating routine, creative, and communication-related managerial tasks.

An integral component of digital readiness is the ability to operate within modern project management software environments (e.g., Asana, Trello, Freelo). An employer representative (R1) critically pointed out that recent graduates, upon entering practice, “*failed to appreciate and exploit the opportunity to use certain modern tools, whether it's things like Asana, ChatGPT, social media tools.”*

The same competence deficit was identified in social media management. Although graduates are classified as “digital natives,” their capabilities often terminate at the user interface level. Employers, however, require deep analytical and strategic understanding. Respondent R3 emphasized that a sports manager must “*simply know how social media works, the mechanisms, the algorithms; this is simply what is important to gain from their studies.”* This finding is further supported by Respondent R17, who remarked that “*the more specialized approach is where it worsens. Those are actually specific tools that serve a particular purpose.”*

### Human dynamics and the soft skills deficit

While technological advancements provide the architecture for operational efficiency, the findings demonstrate that interpersonal dynamics remain the cornerstone of success in sports management. Within this category, analytically coded through nodes such as Leadership, Teamwork or Critical Thinking, the research exposed critical deficiencies in graduates' preparedness to withstand the pressures of the contemporary labor market.

A prominent theme resonating across the interviews with industry representatives is the reluctance of recent graduates to assume accountability for their decisions and projects. Employers observe a growing tendency among the younger generation of sports managers to avoid ambiguity, complex decision-making, and potential confrontations. This professional frustration was aptly summarized by Respondent R8: “*Finding an individual who will actually accept that responsibility and is willing to bear it is, in my opinion, becoming harder and harder.”*

This behavioral pattern is frequently accompanied by a passive approach to task execution, characterized by a rigid, short-term “task-oriented mind-set.” In this context, Respondent R10 critically evaluated recent graduates: “*They only did exactly what they were told and brought virtually nothing extra to the table.”* Several employers associated this pattern with a limited tendency to go beyond formally assigned tasks. In their accounts, recently employed graduates were sometimes described as reliable in completing explicit instructions, but less prepared to identify additional needs, propose improvements, or assume responsibility for less structured problems.

A pivotal factor limiting the professional development and long-term adaptability of graduates is their inability to manage feedback and maintain psychological resilience. The data analysis indicates that the academic environment does not adequately prepare students for high-stress scenarios and constructive criticism. Respondent R10 described this phenomenon with clarity: “*When you criticize something they did, they slowly begin to crumble and end up leaving the job.”*

The graduates themselves acknowledge this deficit, admitting that their university curriculum lacked preparation on “*how to simply not break down when a major problem arises”* (Respondent A3). Furthermore, the findings reveal that graduates experience anxiety regarding the reciprocal process; they exhibit a pronounced difficulty in delivering constructive, objective feedback to their subordinates or peers.

In terms of communication proficiency, employers report a paradox: despite possessing well-developed presentation skills, graduates frequently fail in high-stakes personal negotiations and demanding interpersonal interactions. This is illustrated by the testimony of Respondent R5, who emphasized the necessity of persuasive capabilities: “*You are sitting at the table with them, you have to persuade them, and you simply must know how to do it.”* Instead of engaging in assertive face-to-face or telephonic communication, graduates display a defensive tendency to retreat into the perceived safety zone of text-based, asynchronous online communication, which ultimately hinders effective relationship-building within sports organizations.

### Expectations vs. reality and the experiential learning imperative

The third analytical dimension illuminates a widening theory-practice gap between the conceptual frameworks transmitted within higher education institutions and the agile, fast-paced reality of sports organizations. Employers generally concede that graduates possess a solid foundational knowledge base; as Respondent R15 confirmed: “*From a theoretical standpoint, they are well-equipped.”*

However, this theoretical framework is insufficiently integrated with operational practice. Outlining the ideal profile of a contemporary graduate, Respondent R12 specified the need to be: “*Theoretically charged and prepared, but simultaneously practical and possessing a broader perspective.”* A direct consequence of this academic isolation is that newly minted managers enter sports organizations with inflated, often unrealistic financial and operational expectations that quickly collide with market constraints. Consequently, transitioning into professional practice manifests as a form of “reality shock.” Respondent R8 explicitly identified this dynamic as: “*…a clash with the reality of how sports clubs actually function.”* Graduates frequently propose highly idealized, textbook solutions, only to subsequently discover that such approaches are unviable due to the resource limitations or structural specificities of real-world sports entities.

The analysis reveals a profound consensus on both sides of the labor market (supply and demand) that the only viable bridge to overcome this systemic barrier is the extensive integration of the experiential learning paradigm into the university curriculum, primarily through long-term internships and mandatory practical placements. Retrospectively, the graduates themselves identify practical experience as the most valuable component of their degree program. As Respondent A2 acknowledged: “*I think that is an irreplaceable experience. It gives a person more than certain courses do.”*

Within the educational ecosystem, structured practice serves as a vital regulatory mechanism; it tempers students' unrealistic expectations, enables them to decode the implicit interpersonal and political dynamics within organizations, and acts as the primary accelerant for securing full-time employment within the sports sector.

### Career sustainability: international scope, intercultural competencies, and lifelong learning within the industry 5.0 twin transition

The fourth major thematic category emerging from the multi-perspective data analysis concerns the long-term sustainability of managerial careers within an inherently evolving, globalized sports ecosystem. The advent of Industry 5.0, characterized by the “twin transition” (simultaneous digital transformation and a return to human-centric, sustainable principles), effectively obliterates the geographical boundaries of the sports market. This shift imposes unprecedented demands on sports managers, specifically requiring a synergy between advanced digital adaptability and sophisticated intercultural communication.

The findings demonstrate that linguistic proficiency and international readiness have transitioned from being distinct competitive advantages to baseline market requirements. Industry representatives strongly agree that the capacity to conduct high-stakes business negotiations and deliver public presentations in a foreign language is a fundamental necessity. English maintains a dominant position in this domain, as succinctly summarized by Respondent R5: “*I believe English is an extremely critical aspect; I view other languages merely as a bonus.”* However, language skills in isolation, devoid of international contextualization, are deemed insufficient by the market. Both employers and graduates issue a powerful appeal for the systematic integration of international mobility and global internships into the higher education curriculum. Reflecting this professional demand, Respondent R14 explicitly advocated: “*I would firmly sustain and promote these international student internships.”*

Beyond mere geographical mobility, digital technologies increasingly connect heterogeneous teams across diverse demographic and social strata, thereby amplifying the necessity of managing complex cultural dynamics. Evidence from the graduate interviews suggests that the capability to navigate organizational diversity and intergenerational friction is becoming paramount for modern sports governance. In this context, Graduate A6 highlighted the ethical dimension of modern sports, emphasizing the mandatory reflection of corporate social responsibility themes: “*Themes such as diversity, equity, and organizational integrity […] are an inseparable part of the modern sports environment.”*

Within the framework of Sport Industry 5.0, proficiency in these socio-cultural challenges is no longer a peripheral soft skill; it represents an essential prerequisite for sustainable, human-centric leadership, ensuring that digital acceleration does not compromise human dignity or inclusivity.

#### Knowledge obsolescence and the lifelong learning imperative

A critical finding directly tied to the volatile dynamics of Industry 5.0 is the exponential rate of knowledge obsolescence. Specialized expertise acquired by students at the inception of their degree program may already be outdated by the time of their graduation. Respondent R5 vividly illustrated this intense technological and procedural velocity: “*Today, they establish a certain curriculum, and in two years, things are completely fifty percent different.”*

As a consequence of this rapid contraction of knowledge half-life, a strict imperative for lifelong learning emerges. To remain resilient, sports managers must possess the digital literacy to independently navigate emerging data systems and cognitive flexibility to continuously upskill. Crucially, however, industry representatives critically observed that despite the abundance of continuous professional development opportunities, recent graduates often exhibit only a superficial willingness to engage deeply with new, complex topics (as noted by R6, R9, R11, and R17).

Long-term survival and upward mobility in the modern sports sector demand more than the passive absorption of information; managers must actively cultivate their professional brand and digital social capital through strategic community networking. Looking back at their transition to practice, graduates evaluated building a network of contacts, both face-to-face and via professional digital platforms like LinkedIn, as one of the most transformative elements of their development. The vital importance of this social capital was epitomized by Graduate A8: “*As for networking—well, I consider that to be the alpha and omega of career success.”*

**Figure 1:**
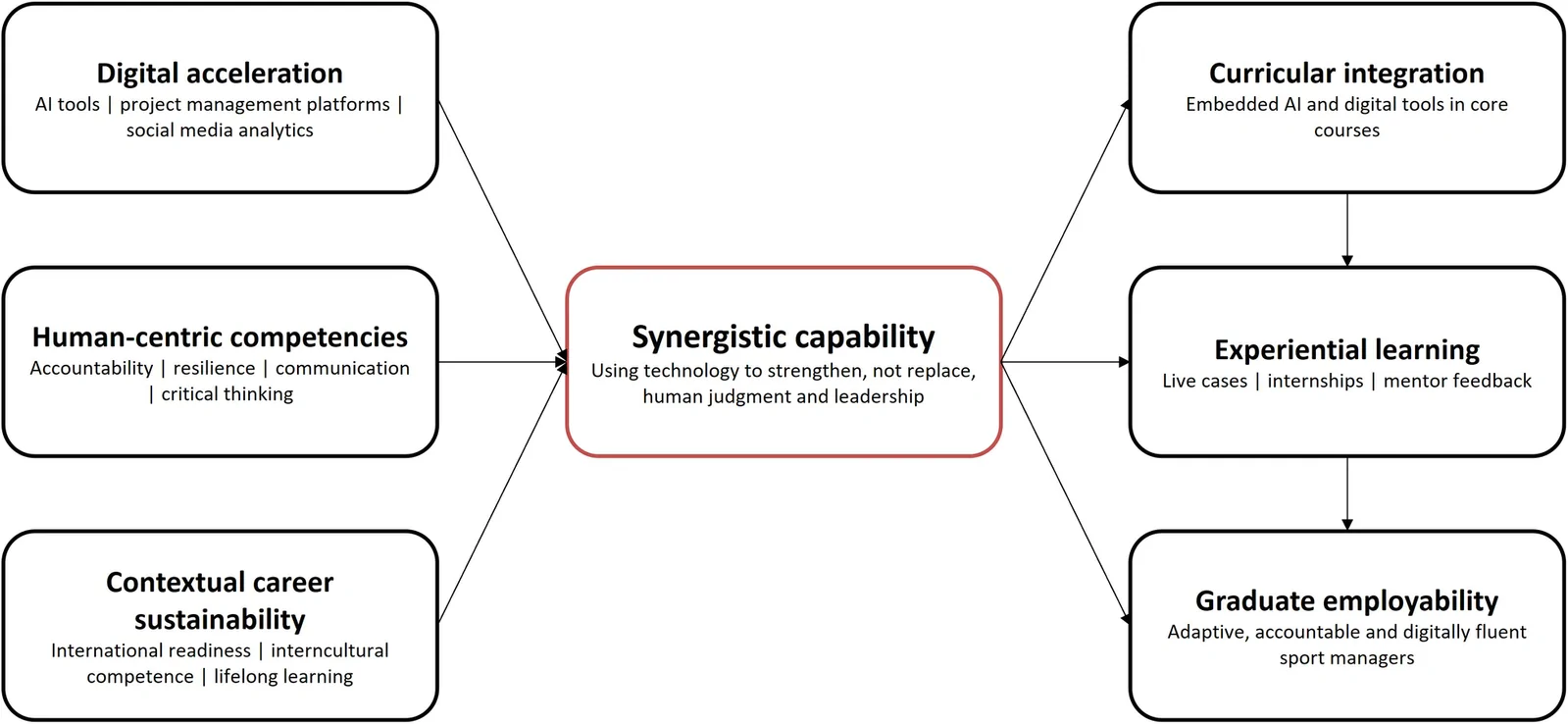
Socio-technical readiness framework for sport managers in the industry 5.0 era. Source: own data.

The empirical data therefore suggest that formal higher education functions as an important baseline, but not as a sufficient condition for long-term readiness in the Sport Industry 5.0 era. True readiness for the Sport Industry 5.0 era resides in an individual's capacity for perpetual adaptability, proactive knowledge acquisition, and the continuous cultivation of intercultural and professional networks. The framework (Figure 1) summarizes four mentioned empirical themes and shows how digital acceleration, human-centric competencies, contextual career sustainability and experiential learning jointly shape graduate employability.

Figure 1 was developed by integrating the four final themes derived from the coding process into a single explanatory structure. The left side of the framework represents the three competency-related conditions repeatedly identified in the empirical data: digital acceleration, human-centric competencies, and contextual career sustainability. These dimensions converge in the central construct of synergistic capability, which reflects the ability to use technology to strengthen, rather than replace, human judgment, communication, and leadership. The right side of the framework translates this readiness logic into educational implications by linking curricular integration and experiential learning to graduate employability. The framework is therefore grounded in the qualitative findings while also providing a conceptual synthesis of how Industry 5.0 readiness can be understood in sport management education.

## Discussion

The present study primarily investigated contemporary labor market demands for sports managers and critically evaluated their professional readiness within the era of Industry 5.0. To guide this empirical inquiry, two research questions were formulated and addressed.

**RQ1:** What competency demands do sports management labor market stakeholders identify in graduates within the context of advancing technological integration?

The findings demonstrate that the labor market no longer considers theoretical knowledge of sports management and marketing sufficient, although employers acknowledge that graduates possess a solid fundamental knowledge base. Representatives of sports organizations now demand a specific, dual-faceted synergy. On one hand, there are rigorous expectations for applied technological competencies, ranging from professional social media management and project management software utilization to, crucially, the ability to implement Artificial Intelligence (AI) tools to optimize workflow efficiency.

Conversely, with advancing digitalization, pressure is paradoxically intensifying on “human dynamics.” Employers identify a critical deficit in graduates' willingness to assume accountability, psychological resilience to stress, assertiveness in negotiation, and capacity to constructively process feedback. Consequently, in response to RQ1, the modern sports labor market demands “human-centric” leaders who can leverage AI and digital systems as performance accelerators, while ensuring that the core of their decision-making and team management remains deeply rooted in interpersonal soft skills and critical thinking (65, 66). This aligns with the foundational philosophy of Industry 5.0 (67), which re-centers the human element at the core of technological systems.

It is important to distinguish between findings that reflect broader and long-standing challenges in sport management education and those that are more directly connected to the Industry 5.0 context. The theory–practice gap, the need for internships, and the importance of communication, leadership, and professional responsibility are not entirely new issues; they have been repeatedly discussed in relation to employability and sport management curriculum development. What appears more specific to the Industry 5.0 perspective is the way these challenges are intensified and reconfigured by digital acceleration, artificial intelligence, and platform-based work. In this context, soft skills are not simply desirable interpersonal attributes but become necessary conditions for the responsible and meaningful implementation of technology.

**RQ2:** What educational model innovations are required to bridge the gap between academic preparation and professional practice, enabling graduates to effectively integrate emerging technologies and artificial intelligence tools into the management of sports organizations?

The findings partly support previous studies emphasizing the mismatch between academic preparation and labor market requirements in sport management education (9, 11, 68, 69). However, they also suggest that this mismatch should no longer be understood only as a lack of practical experience or insufficient curricular responsiveness (70, 71). In the context of Industry 5.0, the gap becomes socio-technical in nature. Graduates are expected not only to know sport management theories or operate digital tools (17, 19, 63), but to combine technological fluency with accountability, adaptive communication, ethical awareness, and resilience under pressure (52). This extends earlier competency-based models (72) by showing that digital and human-centric capabilities cannot be treated as parallel domains of graduate preparedness. Instead, they are mutually dependent: digital tools increase managerial efficiency only when embedded in responsible judgment and interpersonal leadership, while soft skills gain new relevance because they mediate the organizational adoption of technology.

In response to RQ2, this study advocates for a paradigm shift in curricular innovation through the direct integration of digital tools (e.g., AI-driven copywriting, data analytics tools, and platform management such as Asana and Trello) into existing core marketing and strategic management courses. However, this technological integration must be anchored in the extensive deployment of experiential learning. For future sports managers to genuinely internalize the dynamics of Industry 5.0, technological implementation must be practiced under real-world conditions (54, 64, 71, 73). This should be operationalized through live case studies commissioned by industry experts and long-term, structured internships within digitalized sports organizations. Only direct exposure to authentic technological challenges will enable students to fully develop the necessary synergy between digital literacy and human dynamics.

### Implications

**Figure 2:**
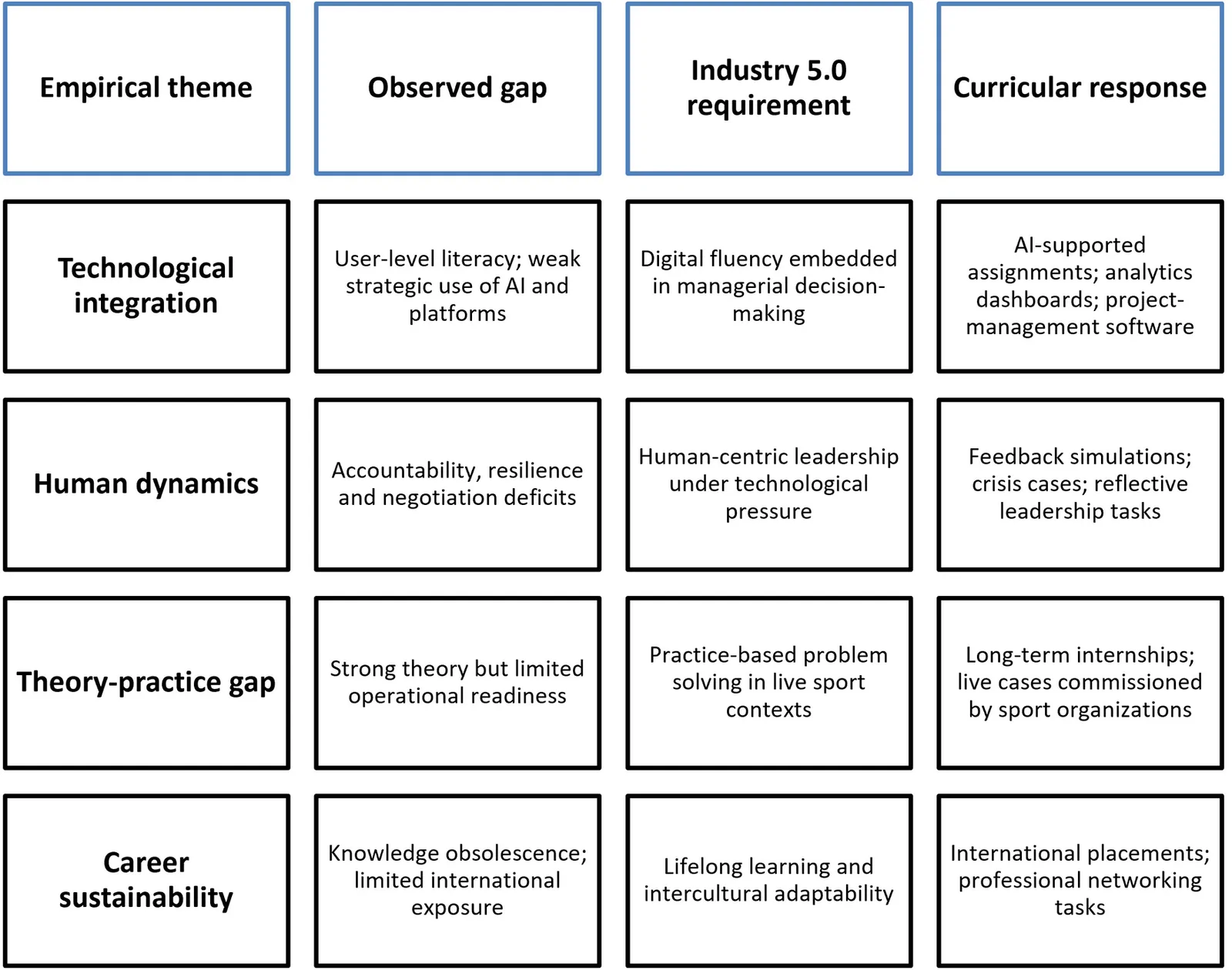
From empirical themes to curriculum innovation. Source: own data.

The findings of this study yield significant theoretical and practical implications for the field of sports management, particularly at the intersection of technological advancement and human resource development. The matrix (Figure 2) translates the four qualitative themes into observable gaps, Industry 5.0 requirements and practical curricular responses. Whereas Figure 1 presents the conceptual logic of socio-technical readiness, Figure 2 translates the empirical themes into concrete educational gaps and curriculum responses relevant for sport management programs.

The theoretical contribution of this study lies in the development of a socio-technical readiness perspective on sport management education. Existing competency models have been highly valuable in identifying relevant managerial, interpersonal, and technical skills, yet they often present these skills as separate domains. The present study advances this discussion by showing that readiness for Sport Industry 5.0 emerges from the interaction between digital acceleration, human-centric competencies, experiential learning, and career sustainability. The proposed framework therefore contributes beyond a descriptive competency list by explaining how these dimensions operate together as a readiness configuration for employability in digitally transforming sport organizations.

From a theoretical perspective (17, 56, 57), this study expands existing sports management paradigms by integrating the dimensions of the Industry 5.0 framework. The results demonstrate that the concept of a “human-centric” approach is not merely a transient theoretical construct (74, 75), but a tangible, empirical demand of the contemporary labor market. Traditional competency models for sports managers must be re-evaluated and redefined.

Rather than conceptualizing AI-driven competencies and digital literacy as isolated, technical skill sets, they must be theorized as complementary elements inherently intertwined with interpersonal dynamics. This study suggests a shift toward a holistic socio-technical competency framework, where advanced digital capabilities (e.g., AI integration, data analytics) and high-level soft skills (e.g., emotional intelligence, cognitive resilience) co-evolve to determine a manager's efficacy in high-tech sports environments.

In practical terms, these findings serve as an urgent call to action for higher education policymakers and curriculum designers. Educational institutions must pivot away from rigid, traditional pedagogical methods, such as rote memorization and passive lecturing, and transition toward agile, experiential learning models. Curricula must undergo comprehensive revision to reflect the volatile dynamics of the industry. This includes embedding mandatory coursework focused on:

- Advanced digital transformation strategies in sports,
- Data-driven online marketing metrics and performance analytics,
- Agile project management methodologies utilizing contemporary software ecosystems (e.g., Asana, Trello).

Furthermore, for sports organizations, these insights highlight a co-responsible imperative to cultivate the next generation of industry leaders. Sports entities can no longer remain passive consumers of academic talent; instead, they must actively participate in talent development by establishing structured internship ecosystems and providing rigorous, high-quality professional mentoring.

For individual practitioners, the findings underscore the necessity of lifelong learning (continuous professional development). In the era of Industry 5.0, the capacity to fluidly adapt to ongoing technological disruption serves as the primary determinant for long-term career sustainability and resilience within the sports management sector.

These findings are directly aligned with the central premise of Sport Industry 5.0: technological innovation in sport should remain human-centric, resilient and socially sustainable. The present study suggests that sport management education must therefore move beyond a narrow “digital skills” agenda. AI, analytics platforms and project management tools become meaningful only when graduates can integrate them into accountable decision-making, interpersonal leadership, ethical communication and reflective professional practice. The contribution of this study lies in showing that the future sport manager is not simply a digitally competent operator, but a socio-technical mediator capable of translating technological acceleration into human and organizational value.

### Limitations and future research directions

While this study provides valuable insights into the intersection of sports management and Industry 5.0 dynamics, several inherent limitations must be acknowledged. First, due to the qualitative nature of the research design, utilizing semi-structured interviews with a sample of 28 participants (*n* = 28), the findings lack generalizability to the broader global population of sports management professionals. Second, the study is geographically and institutionally constrained, as it specifically reflects the socio-economic conditions of one sports labor market and analyzes graduates from a single higher education institution.

Despite the rigorous application of methodological safeguards, including analytical triangulation and strict adherence to qualitative protocols, the interpretation of data remains susceptible to potential researcher subjectivity and positionality (the unique perspective of the researchers influencing data analysis).

These limitations inherently chart promising avenues for subsequent scholarly inquiry. To build upon the qualitative foundation established in this study, future research should deploy extensive quantitative methodologies, such as large-scale cross-sectional surveys, to statistically validate the identified competency gaps across a broader, multi-national sample, thereby enabling cross-cultural comparisons of Industry 5.0 readiness. Additionally, longitudinal studies tracking sports management graduates over the first five years of their professional tenure are highly warranted to map the temporal shift in their perceptions of readiness and to identify which competencies become critical during their career progression.

Future research should also engage more directly with the emerging Sport Industry 5.0 agenda. Building on recent conceptual developments (76), particular attention should be devoted to the interaction between digital transformation, sustainability, organizational resilience, and human-centric innovation in sport organizations. Further studies could explore how sport managers balance technological acceleration with ethical governance, inclusivity, well-being, and long-term social value creation.

A profound research potential also lies in examining the direct causal impact of structured internships on employment velocity and graduate job performance, ideally through a dyadic research approach that simultaneously captures the synchronized perspectives of both the interns and their industry mentors. Given the exponential trajectory of technological progression, future inquiry should scrutinize the granular impacts of generative AI tools on the sports sector, specifically investigating how the integration of large language models and automated analytics algorithms alters the daily workflows, operational routines, and strategic decision-making protocols within both elite and grassroots sports organizations.

## Conclusions

The aim of this study was to comprehensively analyze these emerging labor market demands imposed on sports managers and to uncover the current state of their professional preparedness, focusing specifically on the intersection of digital innovation and the human-centric approach. The findings clearly demonstrate that while current academic programs provide graduates with a solid theoretical foundation, the dynamics of the modern sports industry necessitate a fundamental transformation of their competency profiles. We are entering a period where theoretical knowledge of sport economics and management represents merely a baseline prerequisite, rather than a guarantee of professional success.

The research reveals that future leaders in sports management must be capable of harmonizing two seemingly contradictory yet inherently complementary domains: technological acceleration and human dynamics. On one hand, there is unprecedented pressure on digital literacy, ranging from proficiency in modern project management software to the strategic implementation of artificial intelligence tools, which are becoming an inevitable standard for operational efficiency. On the other hand, technological sophistication loses its significance without a deeply embedded, human-centric approach. Today, more than ever, the labor market demands that managers possess psychological resilience, the capacity to assume accountability for complex decisions, assertive negotiation skills, and the ability to process constructive feedback.

To bridge the gap between academic education and the agile reality of sports organizations, minor, cosmetic curriculum adjustments are no longer sufficient. A structural transformation of educational models is required, one that moves away from traditional lecture-based teaching and the memorization of isolated facts. A viable solution lies in the massive, systemic integration of experiential learning, continuous cooperation with industry experts, and a significant increase in professional placements and international internships directly within study plans. Only direct contact with the sports environment and the resolution of real-world case studies can teach students to effectively implement innovations and prepare them for the socio-cultural specificities of global sports.

The sustainable competitiveness of sports managers in the context of Industry 5.0 thus does not reside in a fixed volume of acquired knowledge, but rather in their long-term adaptability, openness to innovation, and commitment to lifelong learning. However, the same imperative for flexibility and adaptation applies to higher education institutions themselves. If academia is to continue producing successful leaders, it must rapidly respond to these technological and societal trends, thereby ensuring the cultivation of professionals who can utilize digital innovations primarily to enrich the human experience and support the sustainable development of the sports industry.

## Data Availability

The datasets analyzed during the current study are not publicly available due to participant privacy and ethical considerations. Data are, however, available from the corresponding author upon reasonable request.
